# The London Ambulance Scheme

**Published:** 1913-12-06

**Authors:** 


### THE LONDON AMBULANCE SCHEME.

The apathy of the London County Council towards the pressing need of an efficient ambulance service for street accidents has been so frequently commented upon and condemned in The Hospital that the steps taken last week for supplying such a service are naturally gratifying to us. We recognise frankly the difficulty of providing a satisfactory scheme at a reasonable cost, and also that the County Council is in duty bound to study ways and means. But in this matter true economy is not to be found in cheese-paring; and we have felt obliged to criticise the delay which has occurred since this need of London became imperative. However, the long lane has come to a turning at last; and it remains to examine the proposals which are now put forward.

The Metropolitan Asylums Board has undertaken to organise an ambulance service for London under contract with the London County Council: the cost of that part of the scheme which is clearly separable from the Board's existing service is to be defrayed out of the county rate. This is quite a good idea. For the Board has experience in conducting an ambulance service, whereas the Council has not; and at the same time a distinct saving of money will be effected without loss (indeed, probably with gain) of efficiency. The capital expenditure involved is estimated at £18,680, exclusive of the cost of new sites; and the annual expenditure at £17,860. Definite assurance has been given that the new accident and emergency service shall be kept entirely separate and distinct from the infectious diseases service; this is essential, and it is well that the point has been emphasised in the discussion at last Saturday's meeting of the Asylums Board.

The existing ambulance stations of the Board, seven in number, are to be utilised for the housing of accident ambulances also; and ten or twelve small additional depôts are planned, exclusively for accident ambulances. It is thought that these can be distributed in such a way that almost every part of the Metropolis will be within a mile or so of an ambulance station. Each of the new stations will be connected, like the existing ones, by direct private telephone with the head office; and a continuous night and day service will be maintained. This centralisation of the means of communication is probably necessary and unavoidable; but the management of the Board's private exchange in the past has been exceedingly defective, and the new scheme will be a hopeless failure unless this part of it is rendered faultless.

To begin with, one accident ambulance will be provided at each station, with a few held in reserve for breakdowns; electric traction is proposed for these vehicles. A wheeled litter may also be provided at each station. It is noteworthy that the police already possess four hundred of such litters, so nineteen extra will not make an appreciable difference; they will, of course, prove useful in the event of a call reaching a station while the rapid ambulance is in attendance on another case. The minimum staff for each station is placed at two men in constant attendance, both capable drivers and both trained in first-aid. This means an establishment of seven such men per station, which should, we think, prove sufficient to meet all requirements.

For communication, it is proposed to rely on existing telephone facilities, such as call offices and police station wires. Most of the former are not open at night, and to meet this defect either street pillars or additional boxes may, it is stated, be necessary. The disadvantage of the former would be inevitable confusion with the Fire Brigade pillars, and this, in our opinion, will prove a fatal objection. Arrangements are suggested with the General Post Office whereby the telephone operators would put callers through to the head office immediately on receiving a call of "Ambulance" or "Accident" (as may be agreed). It is also to be arranged with the police that the passage of ambulances through the streets shall be facilitated as is that of fire-engines. It is anticipated that thus but a few seconds will elapse from the call to the despatch of the ambulance. The scheme appears to have been carefully and intelligently worked out; and if its operation bears out expectations, a great blessing will have been conferred upon Londoners, and a blot removed from the fair fame of their city. It will be noted that the County Council tacitly acknowledge their financial responsibility for ambulances, a very great change from their former attitude of indolence and neglect.

